# In this issue of *Current Oncology*

**DOI:** 10.3747/co.2007.111

**Published:** 2007-04

**Authors:** M. McLean

This second issue of 2007 finally sees the publication of the first in a series of manuscripts dedicated to the History of Oncology, with a special emphasis, initially at least, on radiotherapeutics. Our European editor, Dr. Richard Mould, writes the second-ever biography of the pioneering work of Pierre Curie (overshadowed to this day by the accomplishments of Marie). Our apologies to Dr. Mould for the delay in the appearance of this manuscript—intended for publication during the 2006 Pierre Curie centennial—in part due to our reorganization and in part to a marked increase in submissions to the journal. This history series will continue through 2007.

When Dr. Laurie Elit and colleagues compared treatment and survival in ovarian cancer over two time periods in Ontario, they discovered that, although age-standardized incidence and mortality rates for this disease have remained stable, advances in management have, it seems, led to a demonstrable improvement in survival for those initially treated with surgery. Their article lays out the data.

On another front, myeloma accounts for only 1.0% of the incidence of all malignancies, but for approximately 13% of all hematologic neoplasms. Until recently, the standard of care was melphalan and prednisone, and although that treatment led to useful improvements in survival, few instances of long-term remission were seen. Now, after nearly 40 years, new drug combinations are being explored, and the goals of the MY.11 trial from the National Cancer Institute of Canada Clinical Trials Group are discussed in this issue.

Our Updates section includes two interesting reviews. Professor Wen G. Jiang from the Metastasis and Angiogenesis Research Group at the Wales College of Medicine discusses hepatocyte growth factor (HGF) and its receptor as potential therapeutic targets; he also raises awareness of the potential of HGF as a molecular imaging tool. Similarly, rather than being simple (and inert) tumour markers, it seems that *CEACAM5* (formerly *CEA*) and *CEACAM6* may represent targets for novel cancer therapies, including cancer vaccines, cellular immunotherapy, radioimmunotherapy, and antibody therapy as delineated by Drs. Carlos Chan and Clifford Stanners from the McGill Cancer Centre.

Finally, Dr. Steven Sagar and colleagues discuss the potential benefits of massage therapy for cancer patients. Their comprehensive review not only presents the possible mechanisms of action that lead to the reported benefits, but also appeals for the use of prospective studies to evaluate the magnitude of those benefits.

